# Prognosis and risk factors for pathological non-response to neoadjuvant chemoimmunotherapy in locally advanced esophageal squamous cell carcinoma

**DOI:** 10.3389/fimmu.2026.1815244

**Published:** 2026-04-29

**Authors:** Jun-Peng Lin, Hui Lin, Hao He, Feng-Nian Zhuang, Wei-Jie Chen, Yu-Jie Chen, Peiyuan Wang, Hang Zhou, Wen-Wei Wei, Peng-Qiang Gao, Shuo-Yan Liu, Feng Wang

**Affiliations:** 1 Department of Thoracic Oncology Surgery, Clinical Oncology School of Fujian Medical University, Fujian Cancer Hospital, Fuzhou, China; 2 Fujian Key Laboratory of Translational Cancer Medicine, Fujian Cancer Hospital, Fuzhou, China; 3 Fujian Provincial Key Laboratory of Tumor Biotherapy, Fujian Cancer Hospital, Fuzhou, China; 4 NHC Key Laboratory of Cancer and Metabolism, Fujian Cancer Hospital, Fuzhou, China

**Keywords:** esophageal squamous cell carcinoma, neoadjuvant immunotherapy, pathological non-response, prognosis, risk factors

## Abstract

**Background:**

Neoadjuvant chemotherapy combined with immunotherapy (NACI) is promising for treating locally advanced esophageal squamous cell carcinoma (LA-ESCC), yet a subset of patients exhibit a pathological non-response (pNR). The prognosis and risk factors for pNR to NACI remain unclear.

**Methods:**

We retrospectively analyzed 253 patients with LA-ESCC who received NACI followed by surgery between 2020 and 2023. The immune checkpoint inhibitors (ICIs), which included pembrolizumab (200 mg), camrelizumab (200 mg), tislelizumab (200 mg), sintilimab (200 mg), nivolumab (200 mg), and toripalimab (200 mg), were administered intravenously once every three weeks. Patients with >50% residual viable tumor cells (Becker TRG3) were classified as having a pNR, while the others were classified as having a pathological response (pR). Survival and recurrence patterns were compared between the two groups. Risk factors associated with pNR and prognosis were identified.

**Results:**

The pNR rate was 39.5% (100/253). Compared with the pR group, the pNR group had significantly worse 3-year overall survival (OS) (58.2% vs. 75.8%, P = 0.001) and disease-free survival (DFS) (46.1% vs. 65.5%, P = 0.001), with higher rates of locoregional (13.0% vs. 4.6%, P = 0.015) and distant recurrence (19.0% vs. 7.8%, P = 0.008). Multivariate analysis confirmed that pNR was an independent risk factor for poor OS (HR 1.926; 95% CI 1.250–2.970; P = 0.003) and DFS (HR 1.899; 95% CI 1.300–2.775; P = 0.001). Lymphovascular invasion (LVI) and perineural invasion (PNI) were independent risk factors for pNR (both P<0.001). Within the pNR subgroup, the PNI and ypN2/N3 stage were independent prognostic factors for poor survival (all P<0.05).

**Conclusion:**

pNR to NACI strongly predicts worse outcomes in LA-ESCC patients. LVI and PNI are key risk factors, with PNI or advanced ypN stage further defining a high-risk pNR subgroup warranting aggressive postoperative management and surveillance.

## Introduction

Esophageal cancer is among the most aggressive diseases and is among the seven leading causes of cancer-related death worldwide (1). Neoadjuvant therapy followed by surgery has become a cornerstone in the management of locally advanced esophageal squamous cell carcinoma (ESCC) (2). Among the various preoperative strategies, neoadjuvant chemoradiotherapy (NCRT) has been widely adopted (3, 4). Despite advancements in treatment methods, the prognosis for patients with ESCC remains unsatisfactory, especially in locally advanced cases, where the 5-year survival rate is less than 20% (5).

Recently, the application of neoadjuvant chemotherapy combined with immunotherapy (NACI) has attracted significant attention as a promising alternative treatment strategy. Multiple clinical trials have shown that NACI is safe and effective for patients with locally advanced esophageal squamous cell carcinoma (LA-ESCC), with favorable therapeutic outcomes (6–8). Our prospective randomized controlled clinical trial revealed that the pathological complete response (pCR) rate in LA-ESCC patients who received NACI was comparable to that in the NCRT group, while the incidence of grade 3 or higher adverse events was only 19.2%, which was lower than the 33.3% reported in the NCRT group. Moreover, a multicenter retrospective study revealed that the 2-year survival rate of patients with locally advanced ESCC who received NACI was significantly better than that of patients who received NCRT (9). Although NACI has shown great potential and can lead to favorable treatment responses, 19.0% to 30.8% of LA-ESCC patients still demonstrate poor or no pathological response to this regimen (>50% residual viable tumor cells) (6, 9). Previous studies have indicated that tumor patients with no response after neoadjuvant therapy have a higher risk of recurrence and a poorer prognosis (10, 11). Chevrollier et al. reported that the prognosis in esophageal cancer patients with no response after NCRT was comparable to that in patients who underwent direct surgery, potentially resulting in unnecessary toxicity and delayed time to potentially curative surgery (12). However, there is currently no reported research on the prognosis in and related risk factors for LA-ESCC patients who show pathological non-response (pNR) to NACI.

Therefore, the purpose of this study was to investigate the prognosis and recurrence patterns of LA-ESCC patients who exhibit pNR after receiving NACI and to identify risk factors associated with pNR, thereby providing important evidence for the development of treatment and follow-up strategies for this population.

## Methods

### Patient selection

**Figure 1:**
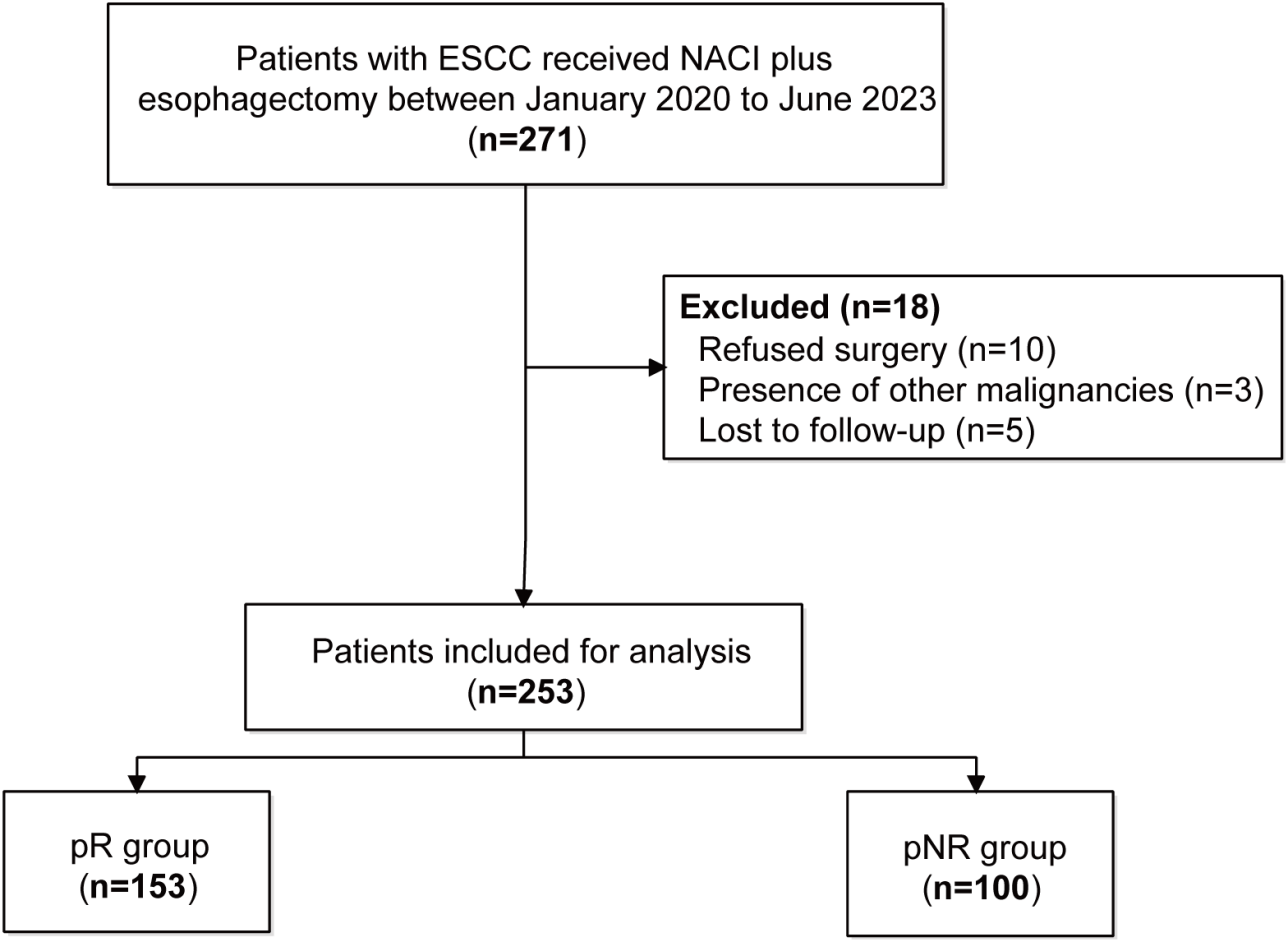
Flowchart of patient selection. ESCC, esophageal squamous cell carcinoma; NACI, neoadjuvant chemoimmunotherapy; pR, pathological response; pNR, pathological non-response.

Patients with locally advanced ESCC who received neoadjuvant immune checkpoint inhibitor (ICI) plus chemotherapy (NACI) and subsequent surgical resection at Fujian Cancer Hospital from January 2020 to June 2023 were identified retrospectively. The inclusion criteria included histological confirmation of ESCC and an age between 18 and 80 years. The exclusion criteria were (1) a diagnosis of another synchronous malignancy, (2) the absence of surgery, and (3) incomplete clinical records or follow-up. After applying these criteria, 253 patients constituted the final study cohort (**Figure 1**). Ethical approval was granted by the hospital’s review board (K2024-042-01), and informed consent was obtained from all the subjects.

### Treatment protocol

Neoadjuvant therapy consisted of 1–4 cycles of combined ICI and chemotherapy. ICIs—pembrolizumab, camrelizumab, tislelizumab, sintilimab, nivolumab, or toripalimab—were given intravenously at 200 mg per dose every three weeks. Chemotherapy followed a 21-day cycle schedule and involved a platinum agent (cisplatin or carboplatin) paired with a taxane (paclitaxel or nab-paclitaxel). All treatment dosages and modifications were at the discretion of the treating physician and were guided by the patient’s clinical condition and body surface area. The operation was performed 4–8 weeks after the completion of the NACI.

### Assessment of postoperative pathology

Postoperative pathological specimens from each patient were evaluated by two experienced pathologists. Pathologic complete response (pCR) was defined as the absence of tumor cells in the entire specimen, including the lymph nodes (ypT0N0M0), whereas major pathologic response (MPR) was defined as <10% viable residual tumor cells in the specimen. The Becker regression criteria were used to evaluate tumor response: TRG1a (no residual tumor cells), TRG1b (<10% residual tumor cells), TRG2 (10%–50% residual tumor cells), and TRG3 (>50% residual tumor cells) (13). In this study, TRG3 were identified as the optimal cutoff value for predicting long-term survival in ESCC patients after NACI by using X-tile software (14). Thus, patients with TRG3 were classified as pNR, while those with TRG1–2 were classified as pathological responders (pR). Lymphovascular invasion (LVI) was defined as cancer cells invading or existing in the blood or lymphatic vessels (15), while perineural invasion (PNI) was defined as the presence of cancer cells inside any of the three layers of the peripheral nerve sheath (16). Tumor staging was performed according to the 8th edition of the American Joint Committee on Cancer (AJCC) guidelines (17).

### Follow-up evaluation

Patients were followed postoperatively according to a defined schedule: evaluations every 3 months for 2 years, followed by every 6 months during the next 3 years, and yearly thereafter. Follow-up included physical examination, serum tumor marker assessment (including carcinoembryonic antigen (CEA), cancer antigen (CA) 19-9, squamous cell carcinoma antigen, and cytokeratin 19 fragment (CYFRA 21-1)), and imaging (primarily enhanced computed tomography (CT), with additional studies such as positron emission tomography (PET)/CT as needed). Annual endoscopy was advised. Overall survival (OS) was measured from surgery to death or last contact. Disease-free survival (DFS) spanned from surgery to the first occurrence of recurrence, death, or last follow-up. Tumor recurrence required radiographic confirmation or biopsy of a suspicious lesion. Recurrence was classified as locoregional (including the esophageal remnant, anastomosis, or regional lymph nodes), distant (involving nonregional lymph nodes or distant organs), or multiple (concurrent involvement of two or more distinct anatomical sites) (18).

### Statistical analysis

Continuous variables are described as the mean and standard deviation (SD) and were analyzed using Student’s t test or the Wilcoxon test. Categorical variables are expressed as frequencies and proportions and were compared with the χ<συπ>^2^ test or Fisher’s exact test. Survival outcomes were estimated with the Kaplan–Meier method and compared between groups using the log-rank test. Univariate and multivariate Cox proportional hazards regression analyses were used to assess prognostic factors for OS and DFS. Significant factors noted in the univariate analysis were subsequently entered into a Cox regression multivariate model using a forward conditional method. Additionally, multivariable logistic regression was subsequently used to identify independent risk factors for pNR. The logistic regression model was evaluated using the Hosmer-Lemeshow goodness-of-fit test, with a P-value > 0.05 indicating adequate calibration. To reduce overfitting and obtain bias-corrected estimates, bootstrap resampling with 1,000 iterations was applied, and 95% confidence intervals were derived from the bootstrap samples. Two-sided *P* values less than 0.05 were considered to indicate statistical significance. Statistical analyses were performed using SPSS^®^ version 22.0 (IBM, Armonk, New York, USA).

## Results

### Patient characteristics

Overall, 253 patients met the inclusion criteria, among whom 48 (19.0%) were female, with a mean age [SD] of 61.1 years [7.3] (**Table 1**). Most patients (74.7%) had an ECOG performance status of 0, with a mean BMI [SD] of 22.0 kg/m^2^ [3.1]. The tumors were most frequently located in the middle esophagus (49.4%), followed by the lower (36.8%) and upper (13.8%) segments. The majority of tumors were clinically staged as cT3 (77.9%) or cN2/N3 (49.0%). Additionally, most patients (79.8%) received ≤2 cycles of NACI. Among all the patients, 153 (60.5%) had pR, and 100 (39.5%) had pNR. Notably, 51 patients (20.2%) achieved pCR, and 103 (40.7%) achieved MPR. The baseline demographic and clinical characteristics were well balanced between the pR and pNR groups, with no statistically significant differences across all preoperative variables (all P>0.05; **Table 1**).

**Table 1:** Baseline demographics and clinical characteristics.

Variable	Overall (n=253) n(%)	pR (n=153) n(%)	pNR (n=100) n(%)	*P Value*
Age, mean (SD), y	61.1 (7.3)	61.5 (7.5)	60.4 (7.0)	0.283
Sex				0.993
Female	48 (19.0)	29 (19.0)	19 (19.0)	
Male	205 (81.0)	124 (81.0)	81 (81.0)	
BMI, mean (SD), kg/m2	22.0 (3.1)	22.2 (3.0)	21.7 (3.3)	0.191
ECOG score				0.497
0	189 (74.7)	112 (73.2)	77 (77.0)	
1	64 (25.3)	41 (26.8)	23 (23.0)	
Comorbidities				0.724
No	179 (70.8)	107 (69.9)	72 (72.0)	
Yes	74 (29.2)	46 (30.1)	28 (28.0)	
History of smoking				0.449
No	144 (56.9)	90 (58.8)	54 (54.0)	
Yes	109 (43.1)	63 (41.2)	46 (46.0)	
History of alcohol consumption				0.761
No	182 (71.9)	109 (71.2)	73 (73.0)	
Yes	71 (28.1)	44 (28.8)	27 (27.0)	
Tumor location				0.105
Upper	35 (13.8)	25 (16.3)	10 (10.0)	
Middle	125 (49.4)	79 (51.6)	46 (46.0)	
Lower	93 (36.8)	49 (32.0)	44 (44.0)	
cT stage				0.474
T2	39 (15.4)	23 (15.0)	16 (16.0)	
T3	197 (77.9)	122 (79.7)	75 (75.0)	
T4	17 (6.7)	8 (5.2)	9 (9.0)	
cN stage				0.266
N0	27 (10.7)	20 (13.1)	7 (7.0)	
N1	102 (40.3)	58 (37.9)	44 (44.0)	
N2/N3	124 (49.0)	75 (49.0)	49 (49.0)	
cTNM Stage				0.492
II	44 (17.4)	28 (18.3)	16 (16.0)	
III	183 (72.3)	112 (73.2)	71 (71.0)	
IVA	26 (10.3)	13 (8.5)	13 (13.0)	
ICIs				0.373
Camrelizumab	79 (31.2)	54 (35.3)	25 (25.0)	
Tislelizumab	67 (26.5)	42 (27.5)	25 (25.0)	
Pembrolizumab	39 (15.4)	22 (14.4)	17 (17.0)	
Toripalimab	31 (12.3)	15 (9.8)	16 (16.0)	
Sintilimab	22 (8.7)	11 (7.2)	11 (11.0)	
Nivolumab	15 (5.9)	9 (5.9)	6 (6.0)	
Neoadjuvant cycles				0.960
≤2	202 (79.8)	122 (79.7)	80 (80.0)	
>2	51 (20.2)	31 (20.3)	20 (20.0)	

pR, pathological response; pNR, pathological non-response; BMI, body mass index; ECOG, Eastern Cooperative Oncology; ICIs, immune checkpoint inhibitors; SD, standardized difference.

The pathological and postoperative characteristics of the pR and pNR groups are shown in **Supplementary Table 1**. Compared with the pR group, the pNR group was more prone to LVI (64.0% vs. 19.6%, P<0.001) and PNI (46.0% vs. 7.2%, P<0.001). However, there were no significant differences between the groups in terms of the number of lymph nodes removed, postoperative complications, or adjuvant therapy (all P>0.05).

### Survival analysis

**Figure 2:**
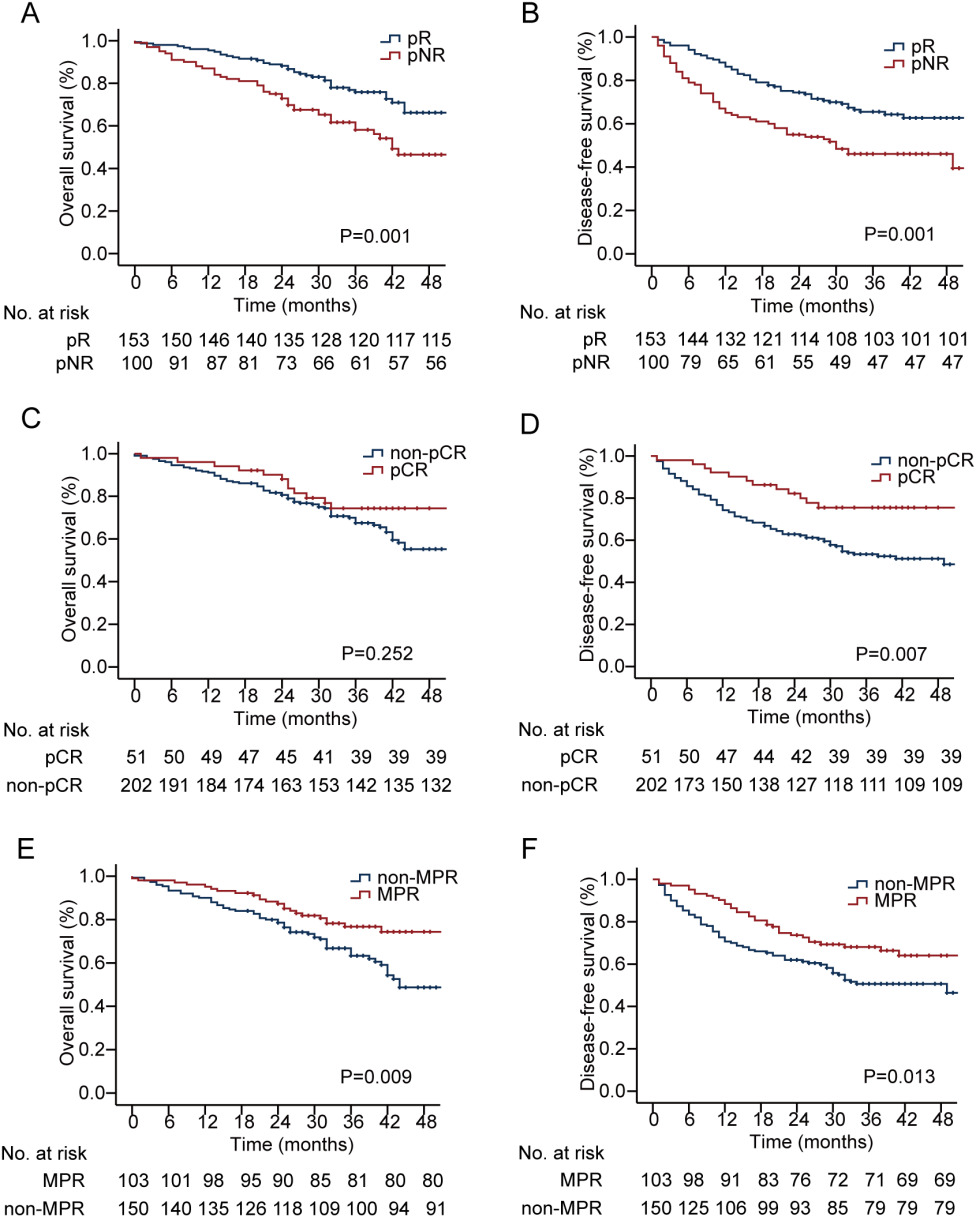
Comparison of **(A)** OS and **(B)** DFS between pR and pNR groups; Comparison of **(C)** OS and **(D)** DFS between pCR and non-pCR groups; Comparison of **(E)** OS and **(F)** DFS between MPR and non-MPR groups. OS, overall survival; DFS, disease-free survival; pR, pathological response; pNR, pathological non-response; pCR, pathologic complete response; MPR, major pathologic response.

The median (IQR) follow-up period was 33.0 (24.0–42.0) months. Kaplan–Meier analysis revealed that patients in the pNR group had significantly worse 3-year OS (58.2% vs. 75.8%, P = 0.001; **Figure 2A**) and DFS (46.1% vs. 65.5%, P = 0.001; **Figure 2B**) than those in the pR group did. We further evaluated the prognostic relevance of two additional pathological response categories. Significant differences in both 3-year OS (76.8% vs. 63.3%, P = 0.009; **Figure 2E**) and DFS (68.1% vs. 50.7%, P = 0.013; **Figure 1F**) were observed between the MPR group and the non-MPR group (both P<0.05). In contrast, while 3-year DFS differed significantly between the pCR group and the nonpCR group (75.5% vs. 53.3%, P = 0.007; **Figure 2C**), no statistically significant difference was found in 3-year OS (74.4% vs. 67.5%, P = 0.252; **Figure 2D**) between these two subgroups.

After adjusting for BMI, pCR, and MPR, multivariate analysis revealed that pNR was the only independent risk factor for OS (HR 1.926; 95% CI 1.250–2.970; P = 0.003; **Table 2**) and DFS (HR 1.899; 95% CI 1.300–2.775; P = 0.001**;****Table 3**).

**Table 2:** Univariate and multivariate cox regression analyses for overall survival.

Variable	Univariable analysis		Multivariable analysis	
	HR (95% CI)	*P Value*	HR (95% CI)	*P Value*
**Age,** y	1.001 (0.972-1.030)	0.969		
Sex				
Female	Reference			
Male	0.823 (0.482-1.404)	0.475		
**BMI, kg/m2**	0.902 (0.836-0.974)	**0.008**	0.909 (0.843-0.980)	**0.013**
ECOG score				
0	Reference			
1	1.097 (0.679-1.773)	0.705		
Comorbidities				
No	Reference			
Yes	0.846 (0.523-1.370)	0.498		
History of smoking				
No	Reference			
Yes	0.835 (0.539-1.294)	0.420		
History of alcohol consumption				
No	Reference			
Yes	0.847 (0.519-1.382)	0.507		
Tumor location				
Upper	Reference			
Middle	0.680 (0.368-1.255)	0.217		
Lower	0.701 (0.371-1.325)	0.274		
cT stage				
T2	Reference			
T3	1.435 (0.738-2.792)	0.287		
T4	1.603 (0.524-4.906)	0.408		
cN stage				
N0	Reference			
N1	0.727 (0.356-1.483)	0.380		
N2/N3	0.766 (0.383-1.532)	0.451		
ICIs				
Camrelizumab	Reference			
Tislelizumab	1.292 (0.715-2.334)	0.396		
Pembrolizumab	1.306 (0.649-2.628)	0.454		
Toripalimab	1.352 (0.648-2.822)	0.422		
Sintilimab	0.927 (0.372-2.313)	0.871		
Nivolumab	2.193 (0.964-4.985)	0.061		
Neoadjuvant cycles				
≤2	Reference			
>2	1.160 (0.688-1.956)	0.577		
pCR				
No	Reference		Reference	
Yes	0.710 (0.393-1.284)	0.257	—	0.692
MPR				
No	Reference		Reference	
Yes	0.538 (0.335-0.865)	**0.011**	—	0.497
pNR				
No	Reference		Reference	
Yes	1.984 (1.288-3.054)	**0.002**	1.926 (1.250-2.970)	**0.003**

pNR, pathological non-response; BMI, body mass index; ECOG, Eastern Cooperative Oncology; ICIs, immune checkpoint inhibitors; pCR, pathologic complete response; MPR, major pathologic response. Bold font indicates statistical significance.

**Table 3:** Univariate and multivariate cox regression analyses for disease-free survival.

Variable	Univariable analysis		Multivariable analysis	
	HR (95% CI)	*P Value*	HR (95% CI)	*P Value*
**Age,** y	0.998 (0.972-1.024)	0.859		
Sex				
Female	Reference			
Male	1.031 (0.634-1.677)	0.902		
**BMI, kg/m2**	0.938 (0.878-1.001)	0.054	—	0.089
ECOG score				
0	Reference			
1	1.123 (0.730-1.729)	0.598		
Comorbidities				
No	Reference			
Yes	0.799 (0.519-1.230)	0.308		
History of smoking				
No	Reference			
Yes	1.103 (0.753-1.615)	0.614		
History of alcohol consumption				
No	Reference			
Yes	1.093 (0.722-1.656)	0.674		
Tumor location				
Upper	Reference			
Middle	0.829 (0.472-1.455)	0.514		
Lower	0.919 (0.514-1.642)	0.775		
cT stage				
T2	Reference			
T3	0.881 (0.529-1.469)	0.627		
T4	0.942 (0.393-2.256)	0.893		
cN stage				
N0	Reference			
N1	0.889 (0.457-1.729)	0.728		
N2/N3	0.992 (0.518-1.899)	0.981		
ICIs				
Camrelizumab	Reference			
Tislelizumab	1.326 (0.794-2.216)	0.281		
Pembrolizumab	1.289 (0.706-2.357)	0.409		
Toripalimab	1.514 (0.809-2.836)	0.195		
Sintilimab	0.978 (0.445-2.148)	0.956		
Nivolumab	1.553 (0.707-3.411)	0.273		
Neoadjuvant cycles				
≤2	Reference			
>2	1.520 (0.982-2.353)	0.060		
pCR				
No	Reference		Reference	
Yes	0.462 (0.258-0.825)	**0.009**	—	0.098
MPR				
No	Reference		Reference	
Yes	0.605 (0.404-0.906)	**0.015**	—	0.703
pNR				
No	Reference		Reference	
Yes	1.899 (1.300-2.775)	**0.001**	1.899 (1.300-2.775)	**0.001**

pNR, pathological non-response; BMI, body mass index; ECOG, Eastern Cooperative Oncology; ICIs, immune checkpoint inhibitors; pCR, pathologic complete response; MPR, major pathologic response. Bold font indicates statistical significance.

### Recurrence patterns

A total of 62 patients (24.5%) experienced recurrence, including 20 patients (7.9%) with local recurrence, 31 patients (12.3%) with distant metastasis, and 10 patients (4.0%) with multiple recurrences. Compared with the pR group, the pNR group had a higher overall recurrence rate (39.0% vs. 15.0%, P<0.001; **Table 4**), particularly in terms of locoregional recurrence (13.0% vs. 4.6%, P = 0.015) and distant recurrence (19.0% vs. 7.8%, P = 0.008). However, there was no statistically significant difference between the two groups in terms of multiple recurrences (6.0% vs. 2.6%, P = 0.307). Notably, the recurrence rate of anastomosis in the pNR group was significantly greater than that in the pR group (6.0% vs. 0.0%, P = 0.003).

**Table 4:** Recurrence patterns in the pR versus pNR groups.

Recurrence type	Total n=253, %	pR n=153, %	pNR n=100, %	*P Value*
**Recurrence**	62 (24.5)	23 (15.0)	39 (39.0)	<0.001
**Locoregional recurrence**	20 (7.9)	7 (4.6)	13 (13.0)	0.015
Anastomosis	6 (2.4)	0 (0.0)	6 (6.0)	0.003
Regional node	14 (5.5)	6 (3.9)	8 (8.0)	0.165
**Distant recurrence**	31 (12.3)	12 (7.8)	19 (19.0)	0.008
Distant lymph node	9 (3.6)	3 (2.0)	6 (6.0)	0.177
Lung	6 (2.4)	2 (1.3)	4 (4.0)	0.340
Liver	5 (2.0)	1 (0.7)	4 (4.0)	0.159
Brain	1 (0.4)	0 (0.0)	1 (1.0)	0.395
Bone	6 (2.4)	4 (2.6)	2 (2.0)	1.000
Pleura	1 (0.4)	0 (0.0)	1 (1.0)	0.395
Multiple organs	6 (2.4)	2 (1.3)	4 (4.0)	0.340
**Multiple recurrence**	10 (4.0)	4 (2.6)	6 (6.0)	0.307

pR, pathological response; pNR, pathological non-response. Bold font indicates statistical significance.

### Risk factors associated with pNR

On the basis of the logistic regression analysis presented in **Table 5**, factors associated with pNR were evaluated. Univariate analysis revealed that pNR was significantly associated with LVI and PNI (both P<0.001), but no significant correlations were observed with other clinical factors, including age, sex, BMI, ECOG score, comorbidities, smoking status, alcohol consumption status, tumor location, cT stage, cN stage, ICIs, or the number of neoadjuvant cycles (all P>0.05). These associations remained statistically significant in the multivariable model after adjustment for other variables, with LVI (OR 5.736; 95% CI 2.926–11.246; P<0.001) and PNI (OR 6.615; 95% CI 2.952–14.825; P<0.001) persisting as independent predictors. And the Hosmer-Lemeshow goodness-of-fit test yielded a χ^2^ value of 5.327 (P = 0.722). To assess the stability of these findings, bootstrap resampling with 1,000 replicates were performed, which showed biases of 0.255 for LVI and 0.251 for PNI, with bootstrap 95% CIs of 3.120-17.484 for LVI and 3.275-32.847 for PNI.

**Table 5:** Univariate and multivariate logistic regression analysis of risk factors for pNR.

Variable	Univariable analysis		Multivariable analysis	
	HR (95% CI)	*P Value*	HR (95% CI)	*P Value*
**Age,** y	0.981 (0.948-1.016)	0.283	0.985 (0.942-1.029)	0.498
Sex				
Female	Reference		Reference	
Male	0.997 (0.524-1.896)	0.993	1.024 (0.406-2.580)	0.960
**BMI, kg/m2**	0.946 (0.870-1.028)	0.192	0.981 (0.881-1.093)	0.734
ECOG score				
0	Reference		Reference	
1	0.816 (0.454-1.468)	0.497	0.874 (0.374-2.044)	0.757
Comorbidities				
No	Reference		Reference	
Yes	0.905 (0.518-1.578)	0.724	1.090 (0.526-2.257)	0.817
History of smoking				
No	Reference		Reference	
Yes	1.217 (0.732-2.023)	0.449	1.337 (0.562-3.181)	0.511
History of alcohol consumption				
No	Reference		Reference	
Yes	0.916 (0.522-1.610)	0.761	0.824 (0.374-2.044)	0.757
Tumor location				
Upper	Reference		Reference	
Middle	1.456 (0.642-3.300)	0.369	1.136 (0.394-3.275)	0.813
Lower	2.245 (0.970-5.194)	0.059	1.918 (0.631-5.828)	0.251
cT stage				
T2	Reference		Reference	
T3	0.884 (0.439-1.779)	0.729	0.718 (0.303-1.699)	0.451
T4	1.617 (0.514-5.089)	0.411	2.798 (0.636-12.301)	0.173
cN stage				
N0	Reference		Reference	
N1	2.167 (0.842-5.581)	0.109	1.608 (0.480-5.389)	0.441
N2/N3	1.867 (0.734-4.745)	0.190	1.162 (0.334-4.043)	0.813
Lymphovascular invasion				
No	Reference		Reference	
Yes	7.289 (4.118-12.901)	**<0.001**	5.459 (2.756-10.813)	**<0.001**
Perineural invasion				
No	Reference		Reference	
Yes	10.997 (5.307-22.788)	**<0.001**	7.295 (3.151-16.890)	**<0.001**
ICIs				
Camrelizumab	Reference		Reference	
Tislelizumab	1.286 (0.648-2.552)	0.472	1.328 (0.538-3.273)	0.538
Pembrolizumab	1.669 (0.757-3.681)	0.204	2.421 (0.853-6.869)	0.096
Toripalimab	2.304 (0.986-5.386)	0.054	2.327 (0.744-7.283)	0.147
Sintilimab	2.160 (0.826-5.646)	0.116	2.890 (0.856-9.762)	0.087
Nivolumab	1.440 (0.462-4.487)	0.529	1.600 (0.389-6.589)	0.515
Neoadjuvant cycles				
≤2	Reference		Reference	
>2	0.984 (0.525-1.845)	0.960	1.126 (0.504-2.517)	0.581

pNR, pathological non-response; BMI, body mass index; ECOG, Eastern Cooperative Oncology; ICIs, immune checkpoint inhibitors. Bold font indicates statistical significance.

**Figure 3:**
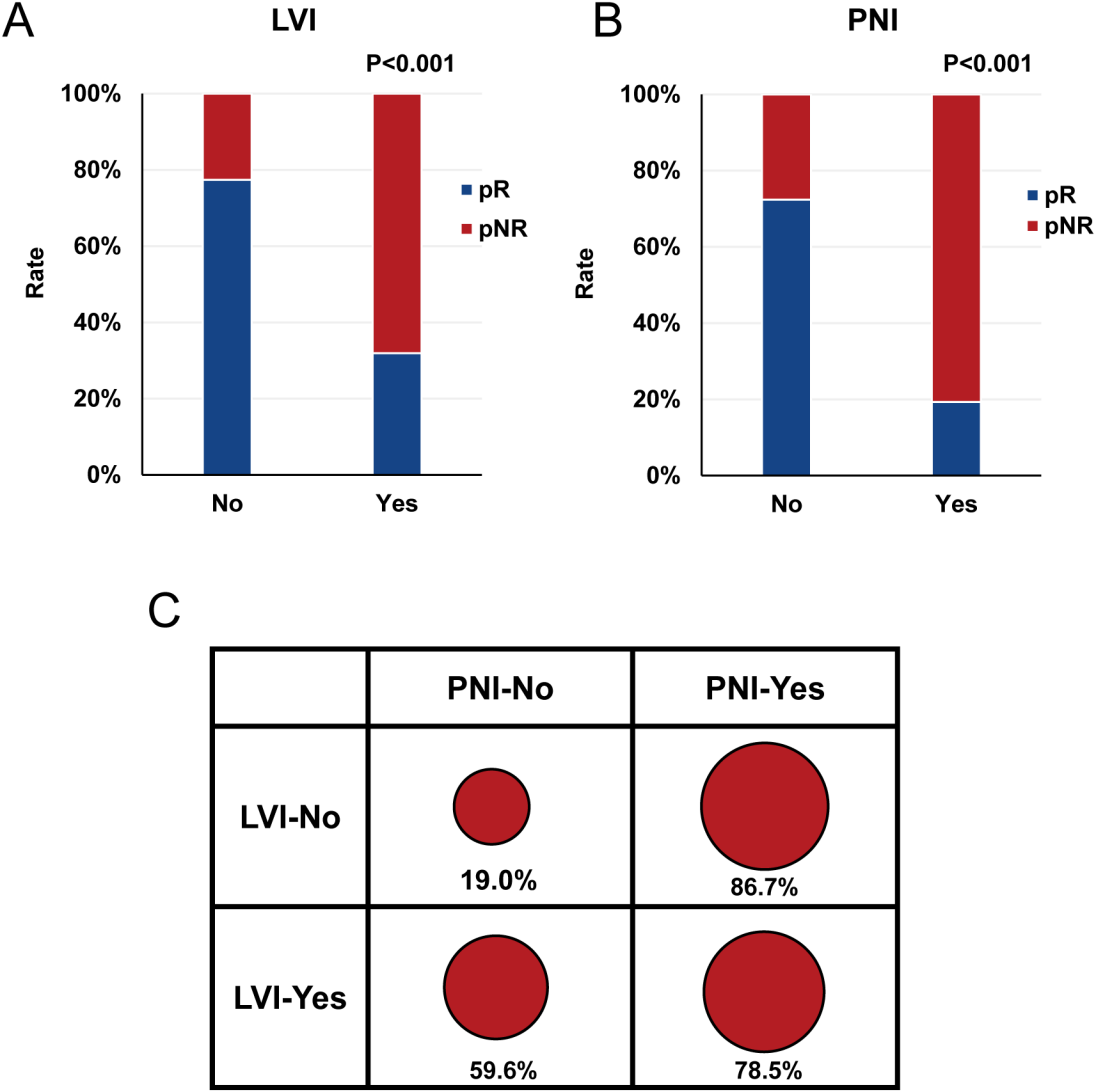
Association of pNR with LVI and PNI **(A-C)**. pNR, pathological non-response; LVI, lymphovascular invasion; PNI, perineural invasion.

Compared with patients without LVI, patients with LVI had a significantly higher pNR (22.6% vs. 68.1%, P<0.001) (**Figure 3**). Similarly, PNI was associated with a markedly elevated pNR (80.7% vs. 27.6%, P<0.001). When grouped by invasion status, the pNR in patients with both LVI and PNI (78.5%) was not higher than that in patients with PNI alone (86.7%).

### Prognostic factors in patients with pNR

In the univariate analysis for OS, an ECOG score of 1, ypT3-4a stage, ypN2/N3 stage, LVI, and PNI were associated with worse OS (all P<0.05; **Supplementary Table 2**). In the multivariate model, an ECOG score of 1 (HR 2.127; 95% CI: 1.118–4.045; P = 0.021), ypN2/N3 stage (HR 2.539; 95% CI: 1.238–5.209; P = 0.011), and PNI (HR 2.235; 95% CI: 1.169–4.272; P = 0.015) remained independent risk factors for OS.

For DFS, univariate analysis revealed an ECOG score of 1, ypN2/N3 stage, LVI, and PNI as significant risk factors (all P<0.05; **Supplementary Table 3**). After these variables were included in the multivariate analysis, only ypN2/N3 stage (HR 2.241; 95% CI: 1.153–4.354; P = 0.017) and PNI (HR 1.774; 95% CI: 1.010–3.156; P = 0.042) were still considered independent prognostic factors.

## Discussion

In recent years, NACI, as a promising neoadjuvant treatment strategy, has garnered widespread attention and has achieved significant progress in the treatment of LA-ESCC. However, previous studies have focused primarily on pCR as the endpoint, and there is currently a lack of relevant research reports on patients with pNR. This study revealed that compared with pCR and MPR, pNR is a stronger independent predictor of poor prognosis and increased recurrence rates in LA-ESCC patients following NACI. Furthermore, the study revealed that LVI and PNI were independent risk factors for pNR. Within the pNR cohort, LVI and PNI were also confirmed as independent risk factors for OS and DFS. These findings provide critical evidence for refining treatment strategy selection and postoperative surveillance for this high-risk patient population.

In this study, 39.5% of LA-ESCC patients had a pNR to NACI, which was consistent with the findings of previous research (6, 19, 20). Specifically, Zhao et al. reported that in LA-ESCC patients treated with neoadjuvant chemotherapy combined with camrelizumab, 45 patients (37.8%) achieved TRG grade 3 (20). Additionally, two other randomized controlled trials demonstrated that approximately 30% of patients with resectable ESCC still experienced tumor regression of less than 50% following NACI (6, 19). Survival analysis revealed that compared with pR patients, pNR patients had significantly inferior long-term survival outcomes, including OS and DFS, which aligns with observations from neoadjuvant therapy studies in other tumor types (11). Furthermore, we observed that while MPR effectively stratified OS and DFS, pCR did not demonstrate a statistically significant prognostic value for OS. Multivariate analysis further confirmed that pNR was an independent risk factor for OS and DFS, whereas MPR and pCR were not. Consequently, pNR may serve as a critical surrogate endpoint for evaluating survival outcomes in LA-ESCC patients following NACI.

In terms of recurrence patterns, the overall recurrence rate in the pNR group was 39.0%, which was significantly higher than that in the pR group, with locoregional recurrence and distant metastasis rates markedly elevated. Notably, anastomotic recurrence was significantly higher in the pNR group than in the pR group, which may be explained by several mechanisms. First, although margin status was a known risk factor (21), only margin-negative patients were included, ruling out this explanation. Second, pNR tumors are less responsive to NACI and exhibit more aggressive biology, as evidenced by higher LVI and PNI rates, which may promote tumor cell persistence at the anastomotic site. Third, chemotherapy relies on adequate blood supply; poorly perfused areas such as the perianastomotic region may have insufficient drug exposure, allowing local tumor cell survival. Collectively, these factors may contribute to the elevated risk. Thus, intensifying local radiotherapy may be considered for pNR patients to reduce anastomotic recurrence. Concurrently, pNR patients also face a significantly increased risk of distant metastasis, which aligns with conclusions from prior basic research indicating a synergistic and mutually reinforcing relationship between tumor treatment resistance and the metastatic process (22). Consequently, for this pNR patient population, intensifying systemic adjuvant therapy postoperatively may contribute to improved survival outcomes.

Owing to the high recurrence rate and poor prognosis in patients with pNR, identifying clinicopathological factors closely related to pNR is crucial for risk stratification and guiding subsequent treatment decisions. In this study, we found no significant correlation between pNR and multiple preoperative clinical factors, including age, sex, smoking status, alcohol consumption, tumor location, cT stage, cN stage, or the number of neoadjuvant cycles. Since the diagnosis of LVI and PNI primarily relies on postoperative histopathological examination, preoperative diagnosis is extremely challenging (2, 23). Therefore, this study utilized postoperative pathological data for analysis and revealed that LVI and PNI are independent risk factors for pNR. Notably, regardless of the presence of LVI, patients with PNI had a pNR of approximately 80%, suggesting that perineural invasion may be strongly associated with the pNR. Since LVI and PNI reflect the more aggressive biological behavior of tumors, characterized by tumor cell infiltration into microvascular and lymphatic networks or diffusion along nerve sheaths to form micrometastatic foci (24, 25), these features may contribute to reduced sensitivity to the cytotoxic and immunomodulatory effects of NACI. Recently, with advancements in artificial intelligence technology (23, 26), accurate preoperative predictions of LVI and PNI based on histopathological and radiomic features have become feasible. This will help identify patients with pNR to NACI before treatment and provide crucial evidence for the adjustment of individualized treatment strategies.

To further guide postoperative management for patients with pNR, this study analyzed prognostic factors within this population. Multivariate analysis revealed that ypN stage and PNI are key factors influencing the OS and DFS in pNR patients, which is consistent with the findings of previous studies (9, 27). A multicenter retrospective study revealed that after neoadjuvant therapy for ESCC, ypN stage (rather than ypT stage) was an independent risk factor for survival (9). Wang et al. also demonstrated that ypN stage was significantly associated with early recurrence after NACI for ESCC (27). Moreover, PNI, a pathological process characterized by tumor cell infiltration along nerve sheaths that facilitates local and distant spread, has been confirmed in multiple studies to be closely related to long-term survival following neoadjuvant therapy for esophageal cancer (28, 29). In summary, this study identifies a particularly high-risk subgroup within the pNR population, namely patients with either PNI or ypN2/N3 stage, who exhibit an exceptionally elevated risk of recurrence and mortality. For such high-risk patients, more aggressive adjuvant therapy and surveillance strategies are recommended postoperatively.

This study has several limitations. First, as a single-center retrospective study, it may be subject to selection bias. And given the limited sample size of this study, future large-scale, multicenter studies are warranted to confirm our conclusions. Second, the follow-up duration might be insufficient to comprehensively evaluate long-term survival differences, particularly regarding the impact of pCR. Nonetheless, a trend favoring OS benefit in the pCR group was observed in the Kaplan-Meier curves. Accordingly, we are continuing follow-up and will conduct a more comprehensive long-term survival analysis in the future. Third, although we identified LVI and PNI as key factors contributing to pNR, these features can be assessed only postoperatively. These findings provide an important foundation for the future development of preoperative predictive models (incorporating imaging or biopsy specimens). For example, these postoperative pathological features could be correlated with preoperative imaging features, biopsy-derived biomarkers, or clinical parameters. Therefore, future research must focus on identifying biomarkers before treatment, such as circulating tumor DNA and radiomic characteristics, to predict the pNR population before NACI, thereby guiding the development of individualized treatment strategies and sparing nonresponders from ineffective treatment and its associated toxicity.

## Conclusion

This study confirms that the pNR following NACI serves as a robust predictor of poor prognosis and increased recurrence risk in patients with LA-ESCC. LVI and PNI were identified as independent risk factors for pNR to NACI. Furthermore, within the pNR population, the PNI and ypN stage were further established as independent prognostic factors. These findings provide important evidence for the early identification of high-risk patients who are likely to develop pNR to NACI and for the formulation of individualized treatment and follow-up strategies for this group.

## Data Availability

The raw data supporting the conclusions of this article will be made available by the authors, without undue reservation.
