# Supplementary material for: Prognosis and risk factors for pathological non-response to neoadjuvant chemoimmunotherapy in locally advanced esophageal squamous cell carcinoma
Source: Front Immunol. 2026 Apr 29;17:1815244. doi: 10.3389/fimmu.2026.1815244 (PMC13167931; doi:10.3389/fimmu.2026.1815244)
Supplement: Supplementary file 1 [file Table1.docx]

**Supplemental Table 1.** Pathology and postoperative characteristics of patients between the pR and the pNR

| Variables | Overall (n=253) n(%) | pR (n=153) n(%) | pNR (n=100) n(%) | *P Value* |
| --- | --- | --- | --- | --- |
| **Resected lymph nodes, mean (SD)** | 41.7 (19.5) | 42.6 (19.6) | 40.4 (19.4) | 0.379 |
| **Lymphovascular invasion** |  |  |  | <0.001 |
| No | 159 (62.8) | 123 (80.4) | 36 (36.0) |  |
| Yes | 94 (37.2) | 30 (19.6) | 64 (64.0) |  |
| **Perineural invasion** |  |  |  | <0.001 |
| No | 196 (77.5) | 142 (92.8) | 54 (54.0) |  |
| Yes | 57 (22.5) | 11 (7.2) | 46 (46.0) |  |
| **ypT stage** |  |  |  | <0.001 |
| T0 | 55 (21.7) | 55 (35.9) | 0 (0.0) |  |
| T1 | 43 (17.0) | 32 (20.9) | 11 (11.0) |  |
| T2 | 44 (17.4) | 33 (21.6) | 11 (11.0) |  |
| T3-4a | 111 (43.9) | 33 (21.6) | 78 (78.0) |  |
| **ypN stage** |  |  |  | <0.001 |
| N0 | 155 (61.3) | 110 (71.9) | 45 (45.0) |  |
| N1 | 57 (22.5) | 28 (18.3) | 29 (29.0) |  |
| N2-3 | 41 (16.2) | 15 (9.8) | 26 (26.0) |  |
| **Postoperative complication** |  |  |  | 0.788 |
| No | 177 (70.0) | 108 (70.6) | 69 (69.0) |  |
| Yes | 76 (30.0) | 45 (29.4) | 31 (31.0) |  |
| **Adjuvant therapy** |  |  |  | 0.955 |
| No | 131 (51.8) | 79 (51.6) | 52 (52.0) |  |
| Yes | 122 (48.2) | 74 (48.4) | 48 (48.0) |  |

Abbreviations: pR, pathological response; pNR, pathological non-response.

**Supplemental Table 2.** Univariate and multivariate cox regression analyses for overall survival in pNR cohort

| Variable | Univariable Analysis | |  | Multivariable Analysis | |
| --- | --- | --- | --- | --- | --- |
|  | HR (95% CI) | *P Value* |  | HR (95% CI) | *P Value* |
| **Age,** y | 0.985 (0.944-1.029) | 0.502 |  |  |  |
| **Sex** |  |  |  |  |  |
| Female | Reference |  |  |  |  |
| Male | 1.072 (0.498-2.308) | 0.858 |  |  |  |
| **BMI, kg/m2** | 0.915 (0.827-1.012) | 0.083 |  |  |  |
| **ECOG score** |  |  |  |  |  |
| 0 | Reference |  |  | Reference |  |
| 1 | 2.478 (1.330-4.616) | **0.004** |  | 2.127 (1.118-4.045) | **0.021** |
| **Comorbidities** |  |  |  |  |  |
| No | Reference |  |  |  |  |
| Yes | 0.872 (0.440-1.725) | 0.693 |  |  |  |
| **History of smoking** |  |  |  |  |  |
| No | Reference |  |  |  |  |
| Yes | 0.658 (0.360-1.203) | 0.174 |  |  |  |
| **History of alcohol consumption** |  |  |  |  |  |
| No | Reference |  |  |  |  |
| Yes | 0.685 (0.338-1.389) | 0.294 |  |  |  |
| **Tumor location** |  |  |  |  |  |
| Upper | Reference |  |  |  |  |
| Middle | 0.901 (0.336-2.416) | 0.836 |  |  |  |
| Lower | 1.043 (0.391-2.781) | 0.933 |  |  |  |
| **yT stage** |  |  |  |  |  |
| T1-T2 | Reference |  |  | Reference |  |
| T3-T4a | 2.798 (1.102-7.105) | **0.031** |  | — | 0.459 |
| **yN stage** |  |  |  |  |  |
| N0 | Reference |  |  | Reference |  |
| N1 | 1.364 (0.621-2.995) | 0.439 |  | 1.691 (0.755-3.790) | 0.202 |
| N2/N3 | 3.361 (1.656-6.823) | **0.001** |  | 2.539 (1.238-5.209) | **0.011** |
| **Lymphovascular invasion** |  |  |  |  |  |
| No | Reference |  |  | Reference |  |
| Yes | 2.255 (1.112-4.572) | **0.024** |  | — | 0.410 |
| **Perineural invasion** |  |  |  |  |  |
| No | Reference |  |  | Reference |  |
| Yes | 2.643 (1.431-4.884) | **0.002** |  | 2.235 (1.169-4.272) | **0.015** |
| **Postoperative complication** |  |  |  |  |  |
| No | Reference |  |  |  |  |
| Yes | 1.593 (0.867-2.925) | 0.133 |  |  |  |
| **Adjuvant therapy** |  |  |  |  |  |
| No | Reference |  |  |  |  |
| Yes | 0.926 (0.511-1.677) | 0.799 |  |  |  |

Abbreviations: pNR, pathological non-response; BMI, body mass index; ECOG, Eastern Cooperative Oncology.

**Supplemental Table 3.** Univariate and multivariate cox regression analyses for disease-free survival in pNR cohort

| Variable | Univariable Analysis | |  | Multivariable Analysis | |
| --- | --- | --- | --- | --- | --- |
|  | HR (95% CI) | *P Value* |  | HR (95% CI) | *P Value* |
| **Age,** y | 0.986 (0.949-1.025) | 0.481 |  |  |  |
| **Sex** |  |  |  |  |  |
| Female | Reference |  |  |  |  |
| Male | 1.079 (0.542-2.146) | 0.829 |  |  |  |
| **BMI, kg/m2** | 0.963 (0.886-1.047) | 0.380 |  |  |  |
| **ECOG score** |  |  |  |  |  |
| 0 | Reference |  |  | Reference |  |
| 1 | 1.834 (1.024-3.283) | **0.041** |  | — | 0.142 |
| **Comorbidities** |  |  |  |  |  |
| No | Reference |  |  |  |  |
| Yes | 0.919 (0.500-1.689) | 0.785 |  |  |  |
| **History of smoking** |  |  |  |  |  |
| No | Reference |  |  |  |  |
| Yes | 0.881 (0.516-1.507) | 0.644 |  |  |  |
| **History of alcohol consumption** |  |  |  |  |  |
| No | Reference |  |  |  |  |
| Yes | 0.909 (0.500-1.653) | 0.756 |  |  |  |
| **Tumor location** |  |  |  |  |  |
| Upper | Reference |  |  |  |  |
| Middle | 1.128 (0.430-2.959) | 0.807 |  |  |  |
| Lower | 1.284 (0.491-3.361) | 0.610 |  |  |  |
| **yT stage** |  |  |  |  |  |
| T1-T2 | Reference |  |  | Reference |  |
| T3-T4a | 1.958 (0.923-4.155) | 0.080 |  | — | 0.515 |
| **yN stage** |  |  |  |  |  |
| N0 | Reference |  |  | Reference |  |
| N1 | 1.602 (0.825-3.111) | 0.164 |  | 1.748 (0.894-3.418) | 0.102 |
| N2/N3 | 2.607 (1.362-4.987) | **0.004** |  | 2.241 (1.153-4.354) | **0.017** |
| **Lymphovascular invasion** |  |  |  |  |  |
| No | Reference |  |  | Reference |  |
| Yes | 2.007 (1.090-3.697) | **0.025** |  | — | 0.490 |
| **Perineural invasion** |  |  |  |  |  |
| No | Reference |  |  | Reference |  |
| Yes | 1.934 (1.125-3.324) | **0.017** |  | 1.774 (1.010-3.156) | **0.042** |
| **Postoperative complication** |  |  |  |  |  |
| No | Reference |  |  |  |  |
| Yes | 1.444 (0.831-2.509) | 0.193 |  |  |  |
| **Adjuvant therapy** |  |  |  |  |  |
| No | Reference |  |  |  |  |
| Yes | 1.233 (0.719-2.114) | 0.446 |  |  |  |

Abbreviations: pNR, pathological non-response; BMI, body mass index; ECOG, Eastern Cooperative Oncology.
